# Link for Injured Kids: A Patient-Centered Program of Psychological First Aid After Trauma

**DOI:** 10.1097/PEC.0000000000000535

**Published:** 2015-11-30

**Authors:** Marizen Ramirez, Maisha Toussaint, Briana Woods-Jaeger, Karisa Harland, Kristel Wetjen, Tammy Wilgenbusch, Graeme Pitcher, Charles Jennissen

**Affiliations:** From the *University of Iowa Injury Prevention Research Center, Department of Occupational and Environmental Health; †Department of Community Behavioral Health, University of Iowa College of Public Health; ‡Department of Surgery, University of Iowa Hospitals and Clinics; §Department of Pediatric Psychology, University of Iowa Children's Hospital; and ∥Department of Emergency Medicine, University of Iowa Hospitals and Clinics, Iowa City, IA.

**Keywords:** trauma, injury, psychological first aid, mental health, adolescents

## Abstract

Injury, the most common type of pediatric trauma, can lead to a number of adverse psychosocial outcomes, including posttraumatic stress disorder. Currently, few evidence-based parent programs exist to support children hospitalized after a traumatic injury. Using methods in evaluation and intervention research, we completed a formative research study to develop a new program of psychological first aid, Link for Injured Kids, aimed to educate parents in supporting their children after a severe traumatic injury.

**Methods:**

Using qualitative methods, we held focus groups with parents and pediatric trauma providers of children hospitalized at a Level I Children's Hospital because of an injury in 2012. We asked focus group participants to describe reactions to trauma and review drafts of our intervention materials.

**Results:**

Health professionals and caregivers reported a broad spectrum of emotional responses by their children or patients; however, difficulties were experienced during recovery at home and upon returning to school. All parents and health professionals recommended that interventions be offered to parents either in the emergency department or close to discharge among admissions.

**Conclusions:**

Results from this study strongly indicate a need for posttrauma interventions, particularly in rural settings, to support families of children to address the psychosocial outcomes in the aftermath of an injury. Findings presented here describe the process of intervention development that responds to the needs of an affected population.

Unintentional injury is the most common type of trauma experienced by children annually, leading to more than 11,000 deaths and more than 280,000 hospital admissions in the United States.^1^ Exposure to a variety of traumatic injuries such as traffic crashes or falls may trigger a number of chronic psychological conditions, of which posttraumatic stress disorder (PTSD) is the most prominent.^2–4^ Prevalence of PTSD symptoms among injured youth ranges from 13% to 32%, depending on the time of assessment.^5^

Many injured trauma patients do not immediately display symptoms of distress while in the hospital setting. Hence, a substantial proportion of injured children with adjustment disorders are undiagnosed, do not seek treatment, and are not referred to psychosocial services.^6^ In rural areas, families of traumatized children face additional challenges in the availability and acceptability of mental health resources.^7,8^ Rural hospitals lack standardized protocols for identifying and referring at-risk traumatized children to mental health services.^9^ Moreover, acceptability of psychological services is lower in rural than in urban settings because of increased stigma and decreased anonymity.^8^

One program that may assist children after trauma is Listen Protect Connect—Model & Teach (LPC), a form of psychological first aid originally developed by school psychologists and social workers to support children after various types of crises, particularly natural disasters and violence.^10–12^ Analogous to physical first aid, psychological first aid “uses interpersonal skills provided by individuals to respond to the psychological consequences of (trauma).”^11^ The steps of LPC are to listen without judgment, protect by helping a child feel safe, connect children with others, model positive behavior, and teach about expected reactions. In a quasi-experiment of 20 students with stressful life experiences including traumatic injury and illness, a modified version of LPC was associated with reduced symptoms of PTSD and depression as well as increased school connectedness and coping.^12^

With these promising results, our team conducted translational research to adapt elements of LPC for the posttrauma care of hospitalized children. We called this new program, Link for Injured Kids, a 2-step skill-based program of psychological first aid developed using a patient-centered approach and parents/guardians as potential interventionists.

The 2 steps, links 1 and 2, were created based on a parent-based intervention in the area of teen driving^13,14^ and our pilot study on LPC.^12^ According to our pilot work, when parents and nurses reported 2 key skills—communication and screening—were valued most in their interactions with traumatized children. Link 1 involves communicating with a child by using open-ended questions and reflections, 2 types of motivational interviewing skills that help increase empathy and create a calm and safe place for traumatized children to share feelings. Link 2 involves linking children with family, friends, and other supports. As part of link 2, parents are taught how to screen for stress using the Kessler-6 Screener (2002), a simple 6-question scale that asks children to rate how sad, nervous, restless or fidgeting, hopeless, frustrated, or worthless a child might feel in the past 2 weeks. This helps the parent understand current levels of distress to assist in decisions for seeking additional psychosocial support.

For this current study, we used formative research methods to create the new link program and identify procedures for future implementation and testing of the program for children with severe injuries admitted into a hospital setting. Similar methods have been used to inform the development of interventions in previous studies.^15–18^ Focus groups with parents/guardians and health care providers of pediatric trauma patients at the University of Iowa Children's Hospital (UIHC) were conducted. As the only level 1 trauma center in the state of Iowa, UIHC serves a substantive proportion of rural families. The goals were to (1) explore the trauma experiences and posttrauma reactions experienced by pediatric patients and their caretakers and (2) obtain feedback on specific intervention materials and implementation procedures to inform the refinement of the intervention.

## METHODS

### Recruitment

To understand the experiences of parents/guardians of injured children, we targeted parents of children ages 10 to 17 years treated at UIHC in the past 12 months for an unintentional injury. The UIHC Trauma Registry System was used to identify eligible parents and collect contact information such as mailing address and telephone numbers. An informational postcard was mailed to parents/caregivers followed by telephone invitation 1 week later to participate in a focus group. A letter of invitation was e-mailed to health care providers who were identified by the pediatric trauma nurse coordinator and the pediatric psychologist. For parents and providers who agreed to participate, a confirmation letter and copy of the intervention materials (manual and pamphlet) were mailed along with instructions to read the documents in advance to provide feedback during the focus group. Parents were provided a $40 gift card, free child care, refreshments, and compensation for mileage to encourage rural parents traveling significant distances to attend the focus group. Health care providers received no compensation for participation.

### Data Collection

Focus group sessions lasted for approximately 1 hour. Verbal informed consent was obtained from each study participant before beginning each focus group. Investigators developed questions before the sessions from which focus group facilitators could guide the discussion with both parents and providers. The first set of questions asked about the various types of trauma experienced by participants' children, the kinds of psychosocial consequences observed, and the resources perceived as accessible and acceptable to families, particularly from rural Iowa. The second set of questions addressed implementation of the program for a future study of hospitalized children with trauma. Participants were asked to provide feedback on the intervention materials, our recruitment and training protocols, and the intervention's applicability to injured children and their families.

### Analysis

All focus group sessions were digitally recorded, transcribed, and saved on a research-designated password-protected server. All transcripts were read by a research team member. Through content analyses, domains that elaborated on family experiences postinjury, the need to address psychosocial impacts, specific input to the manual and pamphlet, and ideas for recruitment and training in the hospital setting were identified. Descriptive patterns consistent with existing domains were identified and allowed for the generation of themes. Results were discussed and reviewed during qualitative research team meetings, and specific refinements to the descriptive themes were generated through consensus.

## RESULTS

Two parent focus groups were conducted with a total of 5 mothers. The mechanisms of injuries sustained by their children were a motor vehicle crash, an all-terrain vehicle crash, crashing while riding a dirt bike, and being struck by a car while walking and riding a bike. The provider focus groups included 3 nurses and a pediatric surgeon in the first focus group, and a pediatric emergency medicine physician and pediatric psychologist in the second focus group. Specific themes identified were (1) emotional reactions at both the hospital and after discharge and (2) support provided to children in the aftermath of their injuries.

## EMOTIONAL REACTIONS POSTINJURY

### Wide Spectrum of Acute Emotional Reactions After Injury

Health care professionals reported a broad spectrum of emotional responses by their patients ranging from “none at all” to “frightened and scared to death.” One provider indicated that it is very difficult to predict how a child might react:

“There is a pretty broad spectrum of emotional reactions … I don't think you can necessarily predict which kids and which kind of injury will require more or less after-care psychologically. I think that is really hard. I've seen some kids that have what I feel is like, holy smokes, that's a big deal, and (they) have no problems at all with adjusting emotionally. Whereas, you can see kids that really don't have as significant of an injury and have a lot of problems.”

Emotional reactions of teenagers included “trying to be more stoic,” whereas others experienced “guilt for the other people involved in the accident.” Similarly, parents described their child's emotional reactions as “scared,” “fearful,” “very much afraid,” and “depressed.” Possible reasons for these reactions were suggested by the parents. Parents described the hospital stay as a significant stressor. One parent reported observing her daughter's hopelessness and frustration in dealing with so many different medical procedures and the uncertainty of what would come next:

“I think my daughter experienced a huge degree of feeling helpless because she couldn't see what (was) … about to happen. There she was on her back at the hospital … wasn't allowed to eat or drink anything in case they had to rush her to brain surgery or something, but she was starving. It turns out she actually had to get an MRI … She was frustrated, and we were frustrated, and we were all still wondering if there was going to be something worse (that) could go wrong.”

### Emotional Reactions at Home Due to Continued Frustrations, Physical Limitations, and Isolation

Emotions displayed after returning home were described by parents as angry, frustrated, and depressed caused by dependency and the feeling of “isolation from friends.” One parent reported,

“He was very angry that he couldn't do the things he wanted to do. He was very frustrated that it takes time to heal and didn't like the fact that he needed help taking a shower and help to go to the bathroom.”

Children sustained injuries that limited their mobility, and hence, living arrangements were made to accommodate them at home. One child's bed was moved to the living area because he could not climb the stairs. Another parent reported that family activities were confined to board games and watching movies to adjust to their child's physical limitations. Children were also reluctant to perform the actual activity that caused their traumatic injuries. For example, according to his mother, one child had difficulties riding the all-terrain vehicle again:

“He did say that he doesn't want to drive it right now, and he knows that spring work is coming and that there is still a farm to run.”

A young girl struck by a car had difficulty understanding why she was injured. She constantly asked the question—“why did I deserve this”—to her parents:

“I think what she felt coming home was the continuation of the frustration about, well, ‘Why did this happen?’ I think she even asked, ‘What did I do to deserve this and that kind of thing.’”

### Difficulties Adjusting to School

All parents agreed that returning to school was a difficult process, although this experience was different for each child. On average, it took 2 to 4 weeks before full participation was achieved. One 16-year-old had challenges during recovery, which was compounded by isolation from friends. Her mother expressed:

“Being a 16-year-old girl and a high achiever at school, she felt isolated from her friends and pressure of having to catch up with her school work. She was out of school for the first week and tried to go back part time, just do a few hours a day. At first, she needed to come home after a couple of hours.”

Two parents reported that teachers tried their best to accommodate their child by giving them “ample time to recover fully” and encouraged them to “return when they were ready.” One parent reported that her son received services from the school counselor. Another parent, in contrast, felt her son's teachers neglected to communicate with her promptly about his poor academic performance.

“It took me until I was sitting at parent-teacher conferences and heard his teachers say, ‘He's failing every class. What are you going to do?’ I had told the teachers before he started (that) if you guys see signs of this, ‘Please let me know.’ And none of them did. Then, they back-pedaled and said that we are not trained to make a diagnosis.”

Parents admitted placing more focus on the physical than on the emotional aspects of the injury. The parent who reported her child failing every class stated,

“I failed my son in that matter. I didn't catch it. I didn't see there was something wrong because it wasn't at the forefront of my mind. I was just trying to manage the day-to-day and everything else got shoved underneath the rug. You focus more on the injury itself more than the psychological.”

## EMOTIONAL SUPPORT AFTER INJURY

### Psychological Support in the Hospital Setting and After Discharge

Providers indicated that psychological support is available in the hospital setting but is a selective process provided only to severe cases, particularly brain injuries. Referrals are described as coming from providers, not parents. Another concern is possible stigma associated with psychological care. When asked if parents know about in-hospital psychological resources, the pediatric psychologist responded,

“Some parents want the help, and some don't. When they hear I'm a psychologist, some people have this idea that I'm some type of head doctor or a shrink … Parents understand (when) it comes from the medical personnel that has referred (them to) us and said, ‘These people are going to help you with this.’

Providers recognized that integrating psychological care into trauma care is possible, and physicians and nurses can play a role particularly during discharge. One physician suggested providing families with a “checklist” of potential posttrauma reactions during recovery. The physician goes on to describe a plan for discharge that might involve provision of local resources, particularly for families who return to their rural communities.

“If they've been hospitalized for an injury, I think you could create a pretty generic (protocol) that could be … automatically or easily pulled up into the electronic discharge … Unfortunately, some (families) are going far away and … I don't know if all of them are aware of mental health resources in their own community.”

Parent focus groups echoed this lack of knowledge about community resources, although one parent did recognize that schools “had a lot of good resources” for connection with professional mental health services. All other parents implied that their child could have benefited from some counseling, but they were unaware of services available within their communities.

### Engaging Parents Into the Postinjury Psychological Care of Their Children

Providers observed among their child patients a tendency to depend on their parents for emotional support. The pediatric surgeon elaborated on the parent-child interaction and how children have displayed cathartic moments with their parents after a traumatic injury. Upon admission, children were “stoically laying there like a little statue.” However, once their parents arrived, their emotions erupted and “they're crying like crazy.” “The parents ask if they've been like this all the while, and no, they have not been like that all the while, only when you came.”

Moreover, when presented with this burst of emotion, parents may understandably be unprepared for the trauma. He indicated,

“The kids are worried because they have (a) little bit of separation anxiety from the parent. So you bring the parent in the room, and it's worse.”

When unprepared for trauma, parents may be unable to provide adequate nurturing. For instance, one provider believed that parents may mark the event as insignificant, whereas others may deny that any real trauma occurred. Yet still, other parents may just not have the skills or ability to understand how to deal with a traumatic event occurring to their child:

“Some might (say), ‘You're ok, you're ok and that it is not that much of a big deal’ … Some parents are also in denial, depending on the circumstance of the injury or accident and about what really happened, and I think some people just don't have the cognitive ability to understand the full impact of what is really going on.”

Empowering parents with the knowledge and skills to deal with a traumatic injury is critical for recovery. As the primary caretakers, parents provide the first level of support to their injured child. In this capacity, effective communication is a critical skill that may influence how parents are able to understand the condition of their injured children. As noted by the pediatric psychologist,

“It depends on the family communication and family relationships that are impacting what it is that the parents are able to understand and/or want to listen.”

She goes on to express the need for parental instruction in methods for communicating with children after trauma:

“I do think that there are some people that need some sort of manual or direct instructions on what to look for following an accident than perhaps other parents do.”

Support from a child's wider family and peer network was also found by parents to promote recovery. One parent expressed the importance of the constant presence of close friends during the recovery period and described this support as a vital component to promote emotional healing.

“The very best thing I could tell any parent is to keep their friends close. As much as their friends can come and spend time with them and keep their life normal, it's really important. Without his buddies, it would have been a lot worse.”

Another parent observed the improved mood of her daughter when surrounded by her friends, “At one point, there were 8 or 9 of them in her hospital room.”

## FEEDBACK FOR LOGISTICS OF STUDY PROCEDURES

We asked parents and providers to discuss the kinds of barriers and enabling factors for implementing and testing a program of psychological first aid in a hospital setting. Providers and parents provided ideas for overcoming barriers and recommendations on how the intervention program may be delivered to parents after a traumatic injury.

### Approach to Recruiting and Training Parent-Child Dyads

All health professionals felt it was important to meet parents face to face, rather than try to recruit patients by telephone, e-mail or letter, to achieve rapport and increase successful follow-up. One parent strongly recommended against training by telephone:

“You're going to struggle (conducting training) on the telephone because when you're on the telephone and you have a child that's injured, ‘Mommy, I got to go to the bathroom!’ or the door bell is ringing, or the TV is on.”

All parents agreed that recruitment would be more successful among admitted patients, and close to discharge would be an ideal time to deliver the intervention and provide parents with all educational materials. There was consensus that parents of admitted trauma cases would generally be receptive to this type of program close to discharge “at that point of hope.” At discharge, one parent indicated,

“(Parents) feel better about their medical plan, and they really crave how am I going to make this okay. People are pretty primed; they're happy that everything is going better.”

Children who receive outpatient or emergency care often spend less than 24 hours at the hospital, representing a short timespan to complete initial contact. Still, parents and providers expressed a strong desire and need for families treated at the emergency department to receive the intervention.

### Feedback to Inform Refinement of Psychological First Aid Program

We asked parents and providers to review drafts of intervention materials and provide feedback. All health professionals were highly receptive to the manual and thought it would be beneficial to all families. It is possible that children may display psychological symptoms, and providing parents with this tool enables them to identify symptoms and seek care if necessary. One pediatric emergency physician noted:

“Developing this booklet of information for parents … is very good. Children may have problems after experiencing trauma, and parents can explore this. They can look at the booklet and see if their child shows any signs of problems or changes from that prior to the injury or, for that matter, from any disease or hospitalization.”

The pediatric psychologist suggested making a revision to the manual that would make it relatable to parents and reduce potential stigma. She suggested adding:

“personal vignettes, stories that capture the situation so that they're not alone. It's not like a weird problem that nobody has, and it's more common than you think.”

The concept of enhancing and building upon parent-child relationships was praised by the pediatric psychologist:

“(The program lets parents) be an expert to their child, and figuring out a way to best supplement their skills and add whatever (you) can to help them help their kid.”

One physician noted he “would like a manual that (he) can give to the family.

Parents, too, were given both a pamphlet and manual to review and provide feedback about content. Materials were well received by parents. One parent exclaimed,

“Amazing, it's awesome! Why didn't I get this at the hospital? I think this information is great because the positive thing is [the manual] really seems to have covered a lot of what they may be feeling. You got a lot of what could possibly be there, so I think that's good.”

Another parent recognized that our program taught parenting skills through communication and education about community resources:

“I think this is a good way to provide parents with information on how to communicate with their child. I think this could also be a community outreach.”

Another parent of a boy injured after a bicycling incident said,

“I think it also encourages better parenting. Even though we're all parents and have that natural function, some of us aren't educated on how you should have a conversation with your child… There are the questions to ask. It educates (us) on what we should be asking and what we should be worried about.”

Overall, parents described that the resources listed at the end of the manual (ie, telephone numbers for local mental health agencies) as most useful.

Finally, parents desired more information about how and when a program like this would be delivered to their child. It was unclear if special time should be reserved to sit and deliver the program to their child or if programmatic elements should be integrated into normal conversations. One parent noted:

“When I was reading through it, I was a little confused…I was wondering if we're supposed to sit down with our children and have a session? I wasn't sure if that was true or whether that would be appropriate rather than just working the awareness in normal conversation.”

Parents came to a consensus on the latter and recommended a short description be included in the manual to describe when to deliver the program. Furthermore, parents were given both a pamphlet and manual, and receiving both may lead to confusion when either or both need to be used. Thus, instructing parents to use both materials as needed—the pamphlet as a quick reference and the manual for in-depth examples and instruction—should be clearly described to parents.

## DISCUSSION

Results from this study strongly indicate a need for posttrauma interventions, particularly in rural settings, to support families of children in the aftermath of injury. The most severe injuries that result in hospitalization particularly in rural settings are of concern because of their psychosocial impacts. An intervention such as Link for Injured Kids could support families and providers in addressing the psychosocial outcomes of severe trauma, as indicated by our qualitative findings.

Unfortunately, the psychological aftermath of trauma tends be neglected, despite the recognition among parents and providers that traumatized youth show a “wide spectrum of emotional reactions” from nothing to being frightened to death, depressed, and guilty. Although some patients may exhibit signs of distress while in the hospital setting because of medical procedures, more often, signs of maladjustment occur after discharge when the injured child is sent back to the community and in recovery. In this study, the most notable experiences of distress were encountered by families during recovery at home, adjusting to lifestyle changes and dealing with challenges in school. These findings are consistent with epidemiologic studies that have documented how trauma can severely impact well-being, quality of life, and school performance.^19–22^

“Stepped collaborative care” is one approach used by Zatzick et al^23^ and Kassam-Adams et al^24^ to support injured patients at risk for psychological sequelae. This model involves health care providers in an assessment of stress and postdischarge case management and has been found to be effective in reducing depression and improving behavioral symptoms among traumatized children.^23–25^ A limitation to this approach is that parents are not well integrated into the model, and there is heavy reliance on the physician to perform the intervention through postdischarge sessions. Sustaining this model in a rural health care setting is questionable especially because many patients may live in communities several hundred miles away from a hospital that might provide services under such a model.

We propose to enhance “Stepped Collaborative Care” with the parent/guardian as the key interventionist with their injured child. We learned in this study that traumatized children depend on their parents for support both during a hospital encounter and afterward. We also learned that effective communication skills, education about posttrauma reactions, and facilitating access to social support systems are critical elements of parent-based posttrauma interventions. Link for Injured Kids incorporates these elements into a brief intervention that involves specific skills in reflective listening and screening. It also has great potential for integration into a health care system that connects patient's caretakers and providers with one another and a larger network of mental health resources. A safety net is created surrounding the child and his or her parent/guardian—the individuals most involved in the day-to-day steps to recovery from trauma.

Through this formative research, we were able to identify and design strategies for a larger scale trial of Link for Injured Kids. Focus groups indicated the feasibility of conducting Link for Injured Kids with in-hospital patients. Participants identified the best windows of time to deliver the intervention close to discharge, and possibly within protocols for discharging patients. Despite the challenges, future research involving emergency department patients were encouraged.

Finally, qualitative data informed the refinement of intervention materials to make them acceptable to traumatically injured children and their caretakers. Intervention materials now include sample conversations to be tailored to each child's traumatic injury, and a pocket card was created to summarize the 2 key steps in link. All materials were reviewed by the UIHC Health Literacy Department to reduce text and assure that the language is at an appropriate reading level.

## LIMITATIONS

Despite attempts to recruit a larger sample of participants, the small number of participants may be considered a limitation. However, “mini focus groups” are considered acceptable and at times ideal, especially in this type of study in which “participants have had intense or lengthy experiences with a topic.”^26^ Our focus groups also had a limited number and type of pediatric emergency medicine providers. Feedback from different types of pediatric emergency medicine providers including pediatric intensive care unit physicians and social workers would be crucial before a program like this would be implemented on a full scale in a hospital setting. Finally, Link for Injured Kids was designed specifically for children hospitalized because of a severe unintentional injury. Its application to other types of trauma has yet to be evaluated.

## CONCLUSIONS

Our formative research that used both qualitative and quantitative methods provided valuable insight to the refinement of Link for Injured Kids and direction for implementation and testing. Using this approach to further develop and refine the program assures that intervention elements are patient centered and acceptable to potential end users (parents and caregivers). This research puts us on solid footing to pursue a large-scale trial of Link for Injured Kids in a hospital setting.
